# Per-oral endoscopic dual myotomy for the treatment of achalasia

**DOI:** 10.1007/s10388-021-00863-9

**Published:** 2021-07-17

**Authors:** Xianglei Yuan, Zhe Feng, Yanshi Zhao, Xianhui Zeng, Liansong Ye, Wei Liu, Bing Hu

**Affiliations:** 1 Department of Gastroenterology, West China Hospital, Sichuan University, No. 37 Guo Xue Alley, Chengdu, 610041 Sichuan China; 2 Division of Gastroenterology, Hepatology and Nutrition, Department of Medicine, Loma Linda University, Loma Linda, USA

**Keywords:** Achalasia, Endoscopic treatment, Myotomy, Dual

## Abstract

**Background:**

Repeat per-oral endoscopic myotomy is occasionally performed for persistent/recurrent symptoms in patients with achalasia, and yields favorable outcomes. We investigated a novel technique, per-oral endoscopic dual myotomy (dual-POEM), where a second myotomy was performed during a single session to augment the efficacy and avoid repeat interventions. The aim of this study was to evaluate its feasibility, safety and efficacy.

**Methods:**

Consecutive patients diagnosed with achalasia who underwent dual-POEM (1/2018–5/2019) were prospectively collected and retrospectively analyzed. Patients with baseline Eckardt score ≥ 9, ≥ 10 years of symptoms, and/or having prior interventions other than myotomy received dual-POEM. The primary outcome was clinical success (Eckardt score ≤ 3). Secondary outcomes were procedure-related adverse events, change in lower esophageal sphincter (LES) pressure, and reflux complications.

**Results:**

Seventeen patients received dual-POEM. Procedure-related adverse events were observed in 2 (11.8%) patients (mucosal injury and pneumonitis). Both were minor in severity. During a median follow-up of 33 months (interquartile range, IQR [31,35]; range, 19–36), clinical success was achieved in 16 (94.1%) patients. The median Eckardt score decreased from 9 (IQR [8, 11.5]; range 7–12) to 1 (IQR [1, 2]; range 0–4) (*P* < 0.001), and LES pressure decreased from 25.8 mmHg (IQR [21.7, 33.5]; range 17.7–46.3) to 7.4 mmHg (IQR [6.3, 10.4]; range 2.2–12.6) (*P* < 0.001). Seven (41.2%) patients developed postprocedural reflux either by gastroesophageal reflux disease questionnaire or esophagitis endoscopically, all successfully treated with proton pump inhibitors.

**Conclusion:**

Dual-POEM preliminarily demonstrated high efficacy with a favorable safety profile in patients with achalasia with predictors of treatment failure.

## Introduction

Achalasia is a primary neurodegenerative disorder of the esophagus characterized by incomplete lower esophageal sphincter (LES) relaxation and absence of esophageal body peristalsis [1]. The pathogenesis of achalasia is incompletely understood; thus, treatment mainly focuses on relaxation or mechanical disruption of the LES to relieve symptoms. Conventional treatment modalities include pharmacologic agents, botulinum toxin injection, balloon dilation and laparoscopic Heller myotomy (LHM) [1]. Inspired by the concepts of natural orifice transluminal endoscopic surgery, per-oral endoscopic myotomy (POEM), creating a submucosal tunnel as the operative space for myotomy, was described and first performed in human patients in 2010 [2]. Subsequently, due to its safety and high efficacy [3, 4], POEM has been used as the first-line treatment for achalasia in many centers across the world.

Despite the remarkable success of POEM, a percentage of patients have persistent or recurrent symptoms afterwards and require additional interventions [5–9]. Although the exact reasons for POEM failure remain largely unknown, an incomplete myotomy (namely insufficient gastric myotomy) is considered to be one of the most likely causes [8, 9]. In addition, some risk factors for POEM failure, including higher pretreatment Eckardt score (≥ 9), long disease duration (≥ 10 years), history of previous interventions, and submucosal fibrosis have also been reported [5, 6, 8, 9]. Multiple studies indicated that repeat myotomy can be performed with favorable outcomes in patients with such risk factors after their initial POEM failure [5, 7, 9, 10]; but it increased the health care costs and delayed the time to remission. To avoid repeat interventions in patients with risk factors for POEM failure, we investigated the performance of a technique, per-oral endoscopic dual myotomy (dual-POEM) [11], where dual myotomy was performed during a single procedure. Here, we conducted a retrospective study and aimed to evaluate the feasibility, safety and efficacy of dual-POEM for patients with achalasia who have risk factors of POEM failure.

## Methods

### Patients

In this retrospective study, consecutive patients with achalasia who underwent dual-POEM from January 2018 to May 2019 at West China Hospital of Sichuan University, were included. We reviewed the clinical data of included patients from our prospectively collected database. Patients without available follow-up data were excluded. This study protocol was approved by the Ethics Committee on Biomedical Research, West China Hospital of Sichuan University. Informed consent was obtained from all involved patients.

### Dual-POEM procedure and follow-up protocol

Patients who met at least one of the following criteria underwent dual-POEM: (1) preoperative Eckardt score ≥ 9; (2) long disease duration ≥ 10 years; (3) and prior interventions other than myotomy. All procedures were performed by a single experienced POEM endoscopist (> 300 POEM procedures). Patients were given a liquid diet for 2 days and fasted for at least 8 h before dual-POEM. Antibiotics were given intravenously 30 min before the procedure to prevent infection. A high-definition endoscope (GIF-Q260J; Olympus, Tokyo, Japan) with a transparent cap (D-201-11802; Olympus) was used. All procedures were performed with a carbon dioxide insufflator (UCR; Olympus), high-frequency generator (VIO 300D; ERBE, Tübingen, Germany), hybrid knife (JET2, APC2; ERBE), and endoscopic clips (HX-610-90; Olympus).

**Fig. 1:**
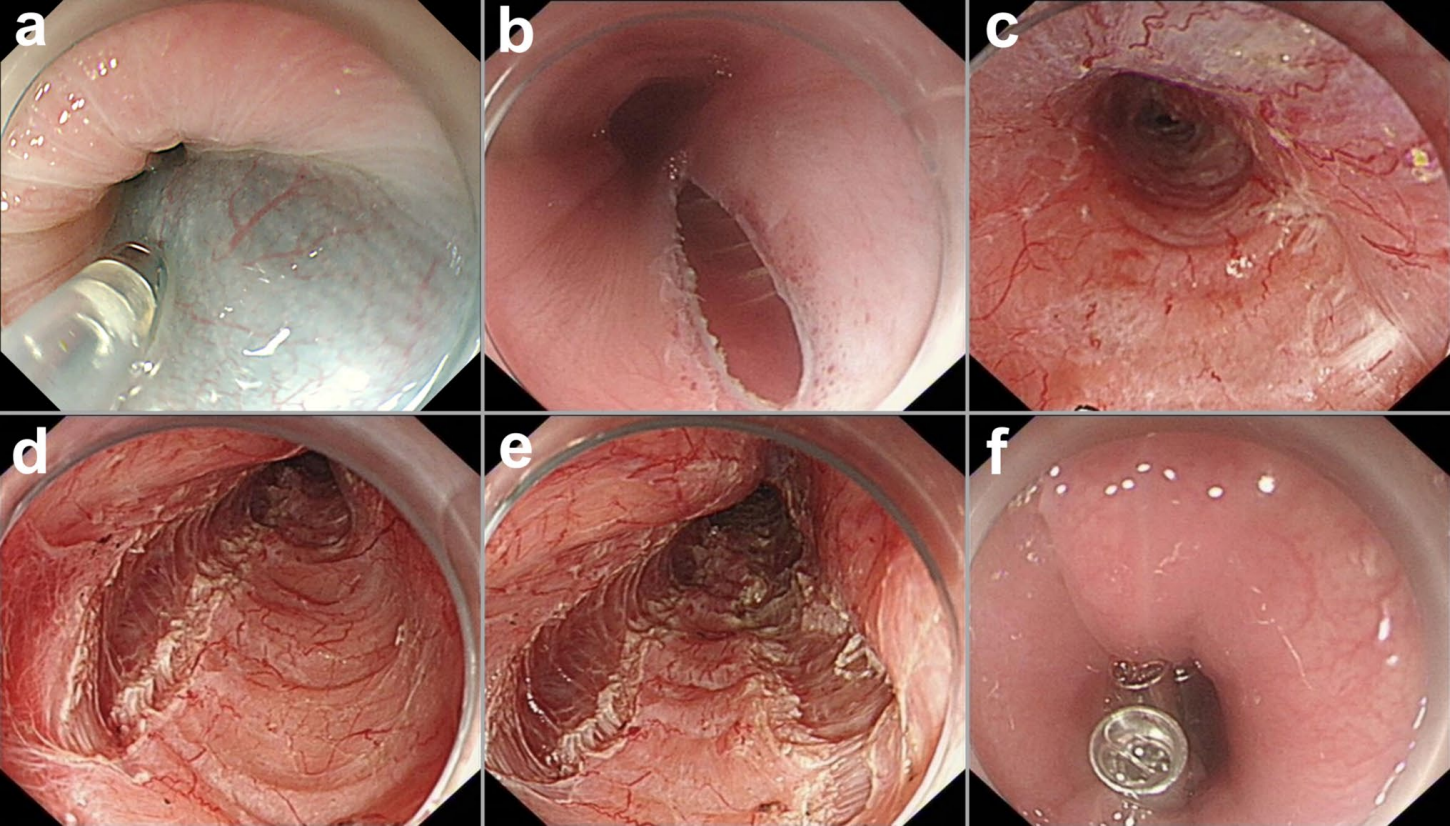
Per-oral endoscopic dual myotomy (Dual-POEM). **a** A submucosal injection with a mixture of methylene blue in saline solution was done. **b** A longitudinal mucosal entry was made. **c** A wide submucosal tunnel, occupying at least half of the esophageal lumen, was created. **d** The first myotomy was performed at the 5–6 o’clock position of the esophagus. **e** The second myotomy was performed at the 1–2 o’clock position of the esophagus. **f** The mucosal entry was closed with endoscopic clips

Patients were placed in a supine position and administrated general anesthesia with endotracheal intubation. A submucosal injection with a mixture of methylene blue in saline solution (0.2 ml in 250 ml) was done approximately 10 cm above the esophagogastric junction (EGJ) at the esophageal wall. A longitudinal mucosal entry with 6–8 mm in length was made. Then, a submucosal tunnel, occupying at least half of the esophageal lumen, was created 3–4 cm below the EGJ. Circular muscle myotomy was performed from 2–3 cm below the incision site down to 2–3 cm below the EGJ. Dual myotomy was performed at the 5–6 o’clock and 1–2 o’clock positions of the esophagus, respectively, which combined the two approaches for standard POEM. Finally, the mucosal entry was closed with endoscopic clips. (Fig. 1). The length of the myotomy was checked by withdrawing the endoscope from the submucosal tunnel into the stomach and observing the color change of the gastric mucosa with a retroflexed view. Complete myotomy could be confirmed by two indicators, including the loss resistance to passage of the endoscope at the EGJ, and observation of the EGJ opening with a retroflexed view at medium insufflation.

After the procedure, patients fasted for 24 h. Intravenous antibiotics were continued for 1–3 days, and intravenous proton pump inhibitor (PPI) was maintained during the hospital stay. A liquid diet was allowed on postoperative days 2–3 if there were no complications, and a soft diet was started within 2 weeks. An oral PPI was administrated for 2 weeks.

The initial follow-up visit was performed at approximately 3 months after dual-POEM. We assessed postoperative Eckardt score, upper gastrointestinal endoscopy, barium esophagography, high-resolution manometry, and reflux complications. Subsequent follow-up visit was then conducted annually to update the Eckardt score and estimate reflux complications. The final follow-up period ended in January 2021.

### Outcomes definition

The primary outcome was clinical success, defined as an Eckardt score ≤ 3 without an indication for retreatment, at follow-up assessment. The Eckardt score was used to rate the severity of achalasia-related symptoms, including weight loss, dysphagia, chest pain, and regurgitation [12]. Recurrence was defined as recurrent symptoms after initial success (Eckardt score > 3). The indication for retreatment was that the patient had recurrence during the follow-up period.

Secondary outcomes included procedure-related adverse events, change in LES pressure, and reflux complications. Procedure-related adverse events were classified as minor or major according to their severity [13]. Reflux complications included symptomatic reflux and reflux esophagitis. Symptomatic reflux was defined as a score equal or greater than 8 in the gastroesophageal reflux disease questionnaire [14]. Reflux esophagitis was graded based on the Los Angeles classification [15]. Achalasia subtype was defined according to the Chicago classification [16]. Procedure time was recorded in minutes from submucosal injection to the end of dual-POEM. Hospital stay was the time that patients spent in the hospital after dual-POEM.

### Statistical analysis

Continuous variables were expressed as mean (standard deviation, SD) or median with interquartile range (IQR) according to their distribution, whereas categorical variables were expressed as frequency or percentage. Median values between baseline and follow-up were compared using Mann–Whitney test. The statistical significance was set to *P* < 0.05. All statistical analyses were conducted using the SPSS version 23.0 (IBM Corp., Armonk, New York, USA).

## Results

### Patient characteristics

During the study period, 17 patients (12 females, 5 males; mean age 43.2 + 11.5 years; range 22–62) with achalasia underwent dual-POEM and were regularly followed up. All of them were included in the final analysis (Table 1). Prior to their dual-POEM, one patient had undergone botulinum toxin injection, and two had received balloon dilation. Twelve (70.6%) patients had pretreatment Eckardt score ≥ 9, and six (35.3%) patients had ≥ 10 years of symptoms. Fifteen patients underwent preprocedural manometry. The most common subtype of achalasia was type II (8/17, 47.1%). The median maximum width of the esophagus was 4.2 cm (IQR [3.7, 5.2]; range 3–6.2), and no patient had a sigmoid esophagus.

**Table 1:** Patient characteristics of 17 patients who underwent dual-POEM

Characteristics	Value
Sex, female/male	12/5
Age, years, mean (SD)	43.2 (11.5)
Duration of symptoms, years, median [IQR] (range)	4 [3, 12] (1–20)
Previous therapy, *n* (%)	
None	14 (82.4)
Botulinum toxin injection	1 (5.9)
Balloon dilation	2 (11.8)
Achalasia subtype, *n* (%)	
Type I	5 (29.4)
Type II	8 (47.1)
Type III	2 (11.8)
No data	2 (11.8)
Maximum width, cm, median [IQR] (range)	4.2 [3.7, 5.2] (3–6.2)

*SD* standard deviation, *IQR* interquartile range

### Procedure-related parameters

All procedures were successfully performed without technical difficulties. The median length of tunnel and myotomy were 10 cm (IQR [10, 12.5]; range 9–14) and 8 cm (IQR [8, 9.5]; range 7–11), respectively. The median procedure time was 35 min (IQR [28, 45.5]; range 21–69). Procedure-related adverse events were observed in 2 (11.8%) patients and were classified as minor. One case of mucosal injury was successfully closed using endoscopic clips at the time of the procedure, without postoperative complications; another case of pneumonitis responded well to conservative treatment. Length of hospital stay was a median of 4 days (IQR [3, 4.5]; range 3–8).

### Follow-up outcomes

**Fig. 2:**
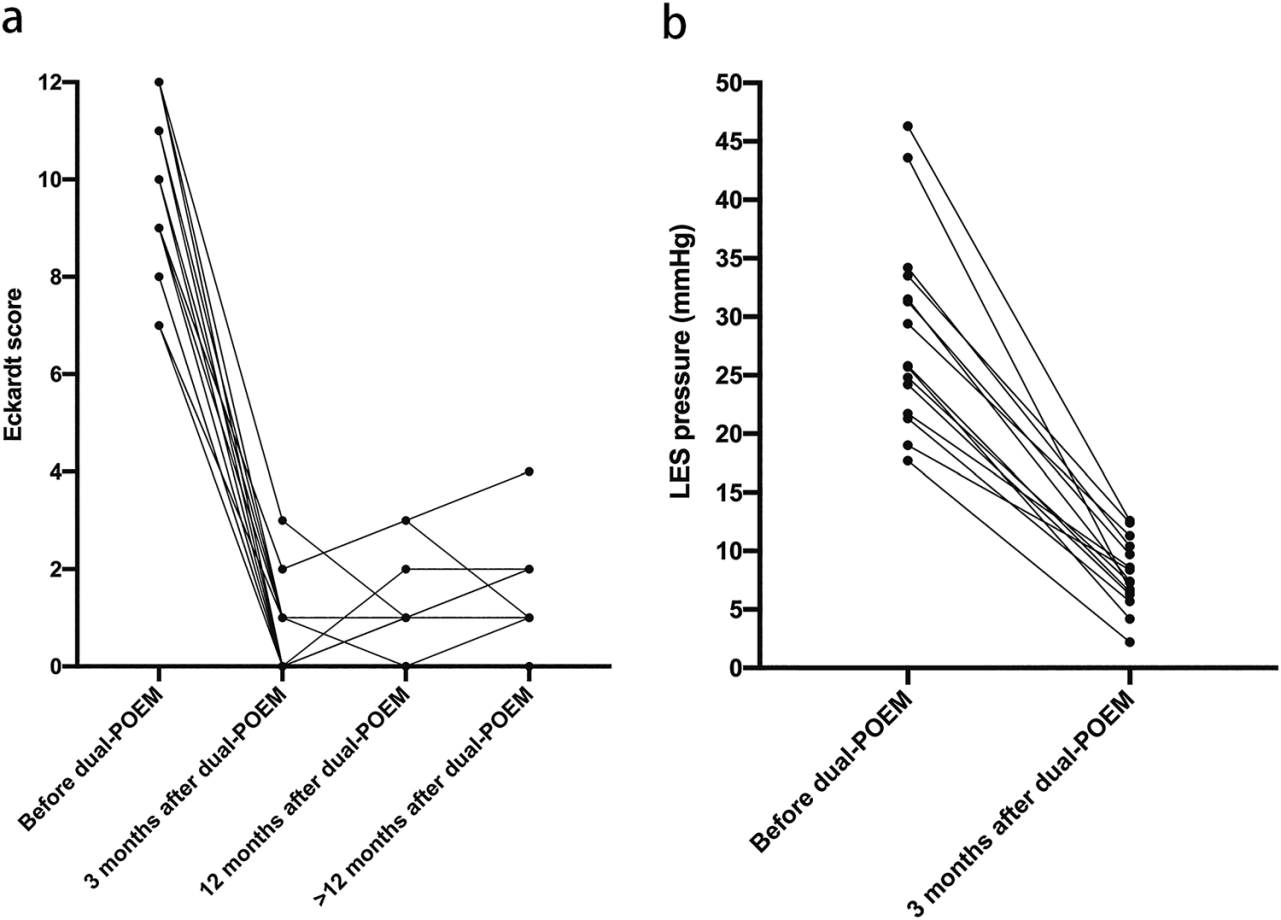
Symptom relief and lower esophageal sphincter (LES) pressure reduction before and after per-oral endoscopic dual myotomy. **a** Eckardt score. **b** LES pressure

**Fig. 3:**
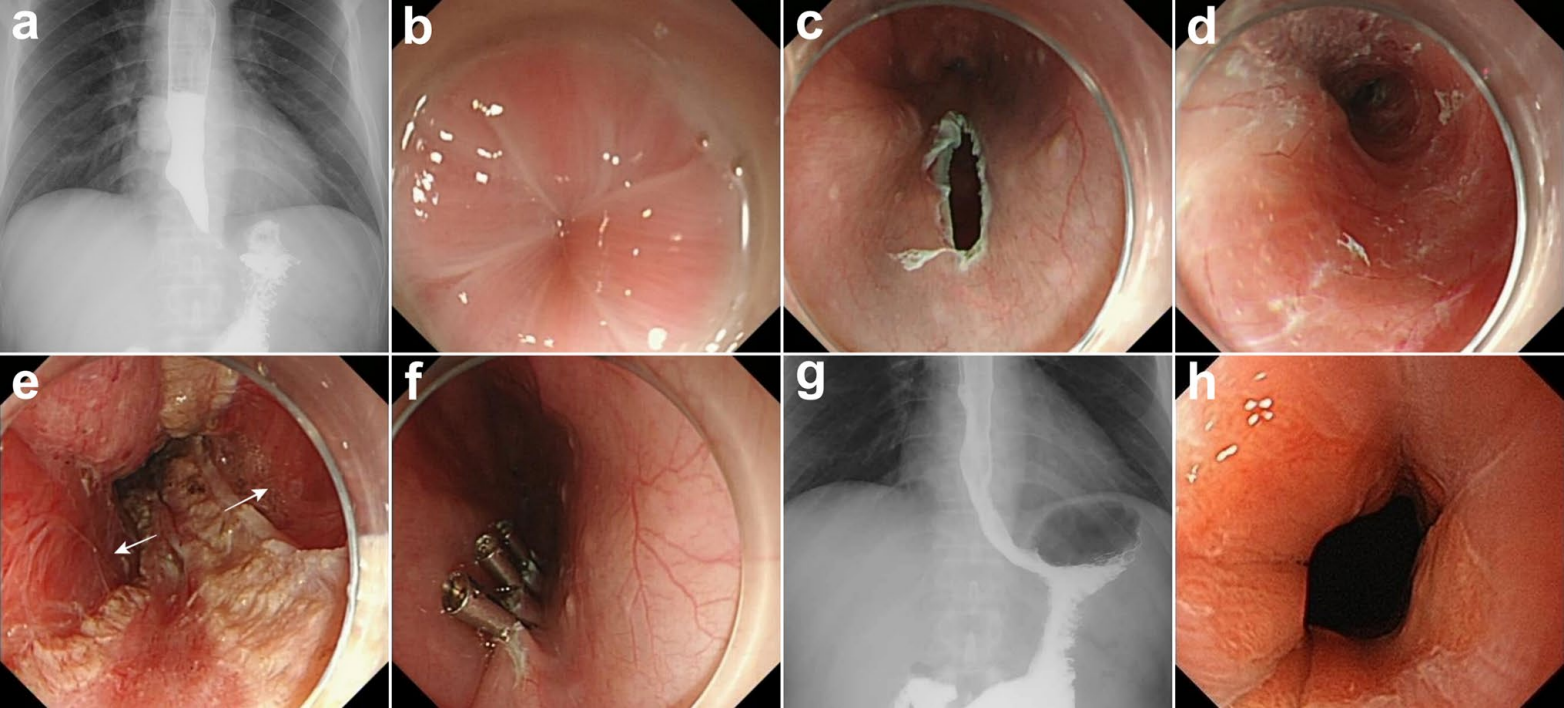
A 36-year-old male with a 11-year history of achalasia. **a** No obvious passage of barium from the esophagus into the stomach. **b** A tightly closed cardia. **c** A longitudinal mucosal entry. **d** Creation of a wide submucosal tunnel. **e** Dual myotomy. **f** Closure of the mucosal entry. **g** Postoperative barium esophagography indicated a rapid passage of barium. **h** Follow-up upper gastrointestinal endoscopy revealed a relaxed cardia

At 3 months after dual-POEM, significant symptom relief and LES pressure reduction were noted in all 17 patients. The median Eckardt score decreased from 9 (IQR [8, 11.5]; range 7–12) to 1 (IQR [0, 1]; range 0–3) (*P* < 0.001) (Fig. 2a) and LES pressure decreased from 25.8 mmHg (IQR [21.7, 33.5]; range 17.7–46.3) to 7.4 mmHg (IQR [6.3, 10.4]; range 2.2–12.6) (*P* < 0.001) (Fig. 2b). At 12-month follow-up, significant symptom relief was continued in all 17 patients, with a median Eckardt score of 1 (IQR [1, 1]; range 0–3). At further follow-up, one patient with achalasia type III developed recurrence. She reported recurrent symptoms with Eckardt score of 4 at 13 months. Subsequently, she underwent a salvage balloon dilation and no recurrence was observed on the 19-month follow-up assessment. During a median follow-up duration of 33 months (IQR [31, 35]; range 19–36), clinical success was achieved in 16 (16/17, 94.1%) patients (Table 2; Fig. 3).

**Table 2:** Follow-up outcomes

Variable	Value
Follow-up duration, months, median [IQR] (range)	33 [31, 35] (19–36)
Reflux complications, *n* (%)	7 (41.2)
Symptomatic reflux only, *n* (%)	1 (5.9)
Endoscopic findings of esophagitis only, *n* (%)	4 (23.5)
Grade A	4
Symptomatic reflux and esophagitis on endoscopy, *n* (%)	2 (11.8)
Grade A	1
Grade B	1
Clinical success (Eckardt score ≤ 3), *n* (%)	16 (94.1)
Recurrence (Eckardt score > 3), *n* (%)	1 (5.9)

*IQR* interquartile range, *POEM* per-oral endoscopic myotomy, *LES* lower esophageal sphincter

Three patients complained of symptomatic reflux, and two of them were found to have reflux esophagitis (one case of Los Angeles classification A and one case of Los Angeles classification B). Four asymptomatic patients had endoscopic evidence of esophagitis (Los Angeles classification A). The rates of symptomatic reflux and reflux esophagitis were 17.6% (3/17) and 35.3% (6/17), respectively. The overall rate of reflux complications was 41.2% (7/17). In these patients, reflux symptoms typically presented at 3 months after dual-POEM, and reflux esophagitis was generally found on the 3-month follow-up endoscopy. These patients were treated with a standard dose of PPI and all achieved clinical response with symptom relief and endoscopic healing of esophagitis.

## Discussion

In this study, we reported a novel technique, dual-POEM, for the treatment of achalasia in the selected group of patients with certain risk factors of POEM failure. No technical difficulties were observed during the dual-POEM procedures; thus, dual-POEM appears to be technically feasible. All patients but one (94.1%) maintained a significant clinical response, achieving posttreatment Eckardt score ≤ 3 at the median follow-up duration of 33 months. No major dual-POEM-related adverse events and no serious reflux complications were observed. Dual-POEM proved to be safe and effective.

Although POEM is considered to be a highly effective treatment for achalasia, POEM failure does occur. Several mid-term studies have shown that the rate of POEM failure ranges from 5.8 to 21.5% [5–8], and increases over time. The efficacy of different retreatments has been extensively studied. Balloon dilation showed a lower clinical successful rate (0–22%) [5, 7], whereas retreatment with POEM or LHM yielded better outcomes (40–100%) [5, 7, 9, 10]. This suggests that repeat myotomy is a more effective remedy after initial POEM failure. Based on this finding, dual-POEM might be a promising method with the potential advantage of avoiding repeat operations. The results of the present series preliminarily confirmed this hypothesis. In our study, in patients having more than one risk factors for achalasia recurrence after initial POEM, clinical success rate was 94.1% during a median follow-up of 33 months. Even though this success rate is close to the ones for standard POEM, the available success rates on standard POEM were obtained from patients with lower pretreatment Eckardt score in general. Our study has looked at a special population with more than one risk factors for treatment failure including higher pretreatment Eckardt score, long disease duration and prior treatments for achalasia, which are usually associated with more severe or refractory disease. A study by Werner et al. [5] has shown that the clinical success rate was around 80% in patients undergoing POEM at 2-year follow-up. Our dual-POEM success rate is better than one reported by Werner; this suggests that dual-POEM may indeed have the advantage in mitigating the risk of recurrence, avoiding a repeat procedure. As for the patient (type III) who developed recurrence, a different pathophysiology from a different manometric subtype is one of the likely explanations [17]. However, given our limited number of patients and relatively short follow-up time, further studies with longer follow-up duration are needed to clarify this phenomenon in type III achalasia.

Regarding the safety of dual-POEM, although the rate of dual-POEM-related adverse events (11.8%) was relatively high when compared with that of standard POEM [18], these adverse events were minor and could be managed conservatively. Mucosal injury was managed successfully with endoscopic clipping. Mild pneumonitis was controlled with administration of antibiotics. Pneumonitis may have been caused by inadequate preoperative esophageal cleaning; thus, preoperative preparation in addition to the use of prophylactic antibiotics and minimizing aspiration should be well executed to reduce the possibility for such infections [19]. In our study, both patients with dual-POEM-related adverse events recovered quickly without apparent deleterious clinical outcomes. Of course, our limited sample size may also have artificially inflated the rate of adverse events. As with standard POEM, gastroesophageal reflux was the major complication of dual-POEM. Previous studies have reported that the rate of symptomatic reflux and reflux esophagitis after standard POEM ranges from 9 to 39.9% [5, 8, 20–22] and 14.8–66% [3, 6, 8, 20], respectively. Comparable results were noted in dual-POEM. In our series, no patients developed reflux esophagitis of more than Los Angeles classification B. All patients who had reflux were successfully treated with PPIs, and none required rescue fundoplication. Dual-POEM does not seem to increase posttreatment reflux disease.

An interesting issue for discussion is on which side dual-POEM should be done. During the standard POEM procedure, two approaches are proposed: anterior and posterior myotomy. Anterior myotomy at the 1–2 o’clock position of the esophagus ensures the minimum curvature of the submucosal tunnel to the stomach and avoids damage to the angle of His (positioned at the 8 o’clock), which is an important natural anti-reflux mechanism [23]. Posterior myotomy at the 5–6 o’clock position enables better alignment of the knife during tunnel creation and myotomy [23]. Investigations indicated that the efficacy and safety of each approach are comparable [23, 24]; thus, endoscopists could choose either approach according to their preferences. Based on these, our dual myotomy was performed at the 1–2 o’clock and 5–6 o’clock positions, respectively. The endoscopist in this study has an extensive experience in POEM; thus, there were no technical difficulties during tunnel creation and dual myotomy. However, the technical feasibility of dual-POEM still needs to be studied among the endoscopists with early POEM experience.

There were some limitations in this study, including its retrospective design, small sample size, a single operator and noncomparative design. Additionally, due to the locally limited accessibility, objective testing such as 24-h esophageal pH measurements was lacking. Although the major limitation seems to be its retrospective design, the data collected from a prospectively collected database supports the validity of the present results.

In conclusion, we report the first case series with favorable results from dual-POEM for the treatment of achalasia in selected patients. Dual-POEM might be a promising option for patients presenting with risk factors of POEM failure. Multicenter, prospective comparative studies with a larger sample size are needed to clarify its indications and to further evaluate its safety and efficacy.
